# Early Adverse Events predict Survival Outcomes in HER2-positive Advanced Breast Cancer Patients treated with Lapatinib plus Capecitabine

**DOI:** 10.7150/jca.41996

**Published:** 2020-03-05

**Authors:** Fang L.I. Ang, Andrew Rowland, Natansh D. Modi, Ross A. McKinnon, Michael J. Sorich, Ashley M. Hopkins

**Affiliations:** College of Medicine and Public Health, Flinders University, Bedford Park, Adelaide, Australia

**Keywords:** adverse events, breast neoplasms, capecitabine, hand-foot syndrome, lapatinib, survival analysis

## Abstract

**Background**: This study aimed to investigate the impact of early adverse events (AE) following the initiation of lapatinib plus capecitabine on the progression-free survival (PFS) and overall survival (OS) outcomes of human epidermal growth factor receptor 2 (HER2) positive advanced breast cancer (ABC) patients.

**Methods**: A secondary analysis of participants treated with lapatinib plus capecitabine, or ado-trastuzumab emtansine in the clinical trial EMILIA was conducted. Cox proportional hazard analysis was used to assess the impact of AE occurring within the first 42 days of lapatinib plus capecitabine therapy on the PFS and OS outcomes of ABC patients.

**Results**: The study included 488 HER2-positive (ABC) patients initiated on lapatinib plus capecitabine. Rash (Hazard Ratio (HR) [95% Confidence Interval (CI)]: Grade 1 = 0.67 [0.46-0.98], Grade 2+ = 0.71 [0.42-1.19]; *p* = 0.046) and hand-foot syndrome (HR [95% CI]: Grade 1 = 0.58 [0.43-0.80], Grade 2+ = 0.61 [0.43-0.86]; *p* = <0.001) occurring within the first 42 days of lapatinib plus capecitabine therapy were significantly associated with improved OS. Conversely, nausea and vomiting occurring within the first 42 days of lapatinib plus capecitabine therapy was significantly associated with worsened OS (HR [95% CI]: Grade 1 = 1.08 [0.82-1.42], Grade 2+ = 1.52 [1.13-2.03]; *p* = 0.027).

**Conclusions**: Rash and hand-foot syndrome occurring early after the initiation of on lapatinib plus capecitabine were significantly associated with improved OS, while early nausea and vomiting was associated with worse OS. In HER2-positive ABC patients initiating lapatinib plus capecitabine, consideration should be given to more closely monitoring patients at risk of nausea and vomiting, while rash and hand foot syndrome are AE associated with improved survival.

## Introduction

Lapatinib (a dual epidermal growth factor receptor (EGFR) and human epidermal growth factor receptor 2 (HER2) inhibitor) and capecitabine (an oral prodrug of 5-fluorouracil) play an important role in later-line treatments of HER2-positive ABC 1-3. This includes patients who experience disease progression on trastuzumab, anthracyclines and taxanes 4, patients with brain metastases (due to lapatinib's ability to penetrate the blood-brain barrier) 5, 6, and patients at risk of cardiac events (lapatinib is less cardiotoxic as compared to trastuzumab) 7.

While lapatinib plus capecitabine is an important treatment for HER2-positive ABC, there is significant variability in survival between patients 8. Correspondingly there has been research to identify markers predictive of survival outcomes from lapatinib plus capecitabine therapy, which ultimately may be used to develop strategies to improve treatment outcomes. Preliminary evidence indicates that early lapatinib plus capecitabine induced AE may be associated with improved survival outcomes. For example, lapatinib plus capecitabine induced rash, hand foot syndrome and diarrhoea have been associated with improved PFS in a small cohort of 76 HER2-positive ABC patients 9. Early lapatinib-related rash (within 42 days) has been significantly associated with OS and a trend towards improved disease free survival in a cohort of 3973 HER2-positive early breast cancer patients receiving adjuvant therapy 10. Hand foot syndrome has also been associated with longer time-to-treatment failure in patients treated with capecitabine (with or without trastuzumab/ irinotecan; n = 98) 11. The aim of this study was to comprehensively assess the association between early AE induced by lapatinib plus capecitabine therapy on the survival outcomes of HER2-positive ABC patients.

## Methods

### Study design and patients

This study analysed individual-participant data collected within the randomized trial EMILIA (NCT00829166) 8, 12. EMILIA included HER2-positive advanced breast cancer patients who underwent prior treatment with trastuzumab and a taxane. Participants were randomly assigned in a 1:1 ratio to either lapatinib plus capecitabine (oral lapatinib 1250 mg per day; oral capecitabine 1000 mg/m^2^ twice per day on days 1 to 14 every 21 days), or to ado-trastuzumab emtansine (3.6 mg/kg intravenously every 21 days) 8, 12. Dose delays, reductions, and discontinuations owing to toxic effects are defined in the EMILIA study protocol 8, 12. Briefly, for capecitabine, the first dose reduction was to 75% of the total daily dose, and the second to 50% of that dose 8, 12. For lapatinib, the first dose reduction was to 1000 mg daily, and the second to 750 mg daily 8, 12. Patients could continue to take lapatinib if capecitabine was discontinued and vice versa 8, 12. If treatment with both drugs was delayed for more than 42 consecutive days, the drugs were discontinued 8, 12.

### Predictor and outcome data

Outcomes evaluated were PFS and OS. PFS was defined from randomization to progression or death from any cause, independently assessed according to the modified Response Evaluation Criteria in Solid Tumours version 1.0 (mRECIST 1.0). OS was defined from randomization to death from any cause, or censored at the date last known to be alive 8, 12.

Grading of AE severity was defined according to the National Cancer Institute Common Terminology Criteria for Adverse Events version 3.0 (CTCAE) 8, 12. AE were evaluated in EMILIA according to Standardized MedDRA Queries, and MedDRA preferred terms were used in this analysis 8, 12. AE that were relatively common early after the initiation of lapatinib plus capecitabine therapy were evaluated for association with OS/PFS in this study. Rash was a combined variable of MedDRA preferred terms rash, exfoliative rash, rash pruritic, rash macular, rash erythematous, rash pustular, rash maculo-papular, rash papular, rash vesicular, and rash generalized.

### Statistical analysis

The primary analysis used a landmark Cox proportional hazard model to investigate the association between survival outcomes and maximum grade of AE at the landmark time. Only AE occurring by the landmark time (first 42 days of lapatinib plus capecitabine therapy) were included, and patients that progressed or died before the landmark time were excluded from the analysis. The landmark point was derived according to a balance of being as early as possible (as early markers of response are more useful, and there is a loss of individuals/power as the landmark time increases due to the event occurring before the landmark) and ensuring enough adverse events had occur before the landmark time (as only adverse events before the landmark count in the analysis technique). Associations were reported as HR with 95% CI, and *p* values (likelihood ratio test). Kaplan-Meier analysis was used assess the effects of AE predictors on OS and PFS.

Sensitivity analysis of identified associations was conducted, including a time-dependent Cox proportional hazard regression to model the association between AE and survival outcomes. Time-dependent Cox proportional hazards analyses adjusted for pre-treatment age, race (white and non-white), ECOG performance status, visceral disease status, months since diagnosis, progesterone receptor status, estrogen receptor status, and any prior anthracycline in any setting, were also conducted.

All statistical analyses were performed with R (version 3.4.3).

## Results

Data from 488 HER2-positive ABC patients initiated on lapatinib plus capecitabine were available. Median follow-up [95%CI] was 45.2 [43.0- 49.6] months. The pre-treatment characteristics of the cohort are summarised in Appendix Table 1. Appendix Table 2 summarises the maximum grade of AE occurring within the first 42 days of lapatinib plus capecitabine therapy, and within the entire follow up period.

There was a significant association between rash occurring within the first 42 days of lapatinib plus capecitabine therapy and improved OS (HR [95%CI] Grade 1 = 0.67 [0.46-0.98], Grade 2+ = 0.71 [0.42-1.19]; *p* = 0.046). There was also a significant association between hand-foot syndrome and improved OS (HR [95% CI]: Grade 1 = 0.58 [0.43-0.80], Grade 2+ = 0.61 [0.43-0.86]; *p* = <0.001). Nausea and vomiting (particularly Grade 2+) was significantly associated with worsened OS (HR [95%CI] Grade 1 = 1.08 [0.82-1.42], Grade 2+ = 1.52 [1.13-2.03]; *p* = 0.027). No statistically significant associations between rash (HR [95%CI] Grade 1 = 0.82 [0.58-1.16], Grade 2+ = 0.68 [0.40-1.17]; *p* = 0.197), hand-foot syndrome (HR [95%CI] Grade 1 = 0.75 [0.56-1.00], Grade 2+ = 1.04 [0.75-1.44]; *p* = 0.106), or nausea and vomiting (HR [95%CI] Grade 1 = 1.11 [0.85-1.45], Grade 2+ = 1.41 [1.05-1.90]; *p* = 0.084) occurring with the first 42 days of lapatinib plus capecitabine therapy and PFS was observed (Appendix Table 3). Further, diarrhoea, decreased appetite, gastrointestinal inflammation, and fatigue/ asthenia were not associated with either of OS or PFS outcomes (Appendix Table 3).

Appendix Figure 1 presents Kaplan Meier estimates of OS and PFS by maximum grade of rash, hand foot syndrome and nausea/ vomiting occurring within the first 42 days of lapatinib plus capecitabine treated ABC patients within EMILIA.

The significant association between rash and hand-foot syndrome with improved OS were confirmed on univariable and adjusted time-dependent Cox proportional hazard regression analysis (*p* = <0.001, Appendix Table 4). The significant association between nausea and vomiting with worsened OS was confirmed on univariable analysis (*p* = 0.044), albeit only a substantial trend towards worsened OS was observed for those experiencing grade 2+ nausea and vomiting on adjustment (*p* = 0.134, Appendix Table 4).

## Discussion

The present study identified ABC patients who experienced rash and hand-foot syndrome within the first 42 days of lapatinib plus capecitabine therapy had improved OS. Conversely, ABC patients who experienced nausea and vomiting within the first 42 days of lapatinib plus capecitabine therapy had worse OS.

Lapatinib-induced rash is an effect of the drugs actions on the ErbB-1 receptor 13. Prior studies have indicated the development of a rash with ErbB-1 inhibitors is associated with better survival outcomes in non-small cell lung and pancreatic cancer patients 14. Further, the identified association herein between lapatinib plus capecitabine therapy induced rash and improved OS is similar to results observed in early breast cancer patients treated with lapatinib; the Neoadjuvant Lapatinib and/or Trastuzumab Treatment Optimisation (NeoALTTO) trial and Adjuvant Lapatinib and/or Trastuzumab Treatment Optimisation (ALTTO) trial demonstrated that early rash was associated with higher rates of pathological complete response, and improved OS respectively 10, 15.

The pharmacological mechanism of capecitabine-associated hand-foot syndrome is still unclear, however preliminary data suggests that the capecitabine-metabolizing enzyme, thymidine phosphorylase, is frequently highly expressed in the palms, leading to elevated local production of 5-fluorouracil, resulting in hand-foot syndrome 16. Similarly, studies have shown that high tumor expression of thymidine phosphorylase is associated with improved survival outcomes 17, 18. Given this information, it is not surprising that prior studies have indicated the development of early hand-foot syndrome (landmark analysis at 3 months) 19 and hand foot syndrome at any time 20 are associated with better survival outcomes in ABC receiving capecitabine, and our study demonstrated hand foot syndrome to be associated with improved OS in ABC receiving lapatinib plus capecitabine.

Age, performance status, prior and concomitant therapies and history of anticipatory nausea and vomiting are known predictors of chemotherapy induced nausea and vomiting 21. The biological rationale by which lapatinib plus capecitabine-induced nausea and vomiting mediates worse OS was not identified within this study but may be a reflection that nausea and vomiting is not related to on-target effects of lapatinib or capecitabine. While the mechanism was not identified, it is not surprising as chemotherapy-induced nausea and vomiting can impact the quality-of-life of cancer patients, and patient-reported outcomes are associated with survival outcomes 22. Given this prior evidence and the observation herein that ABC patients who experienced nausea and vomiting within the first 42 days of lapatinib plus capecitabine therapy have worse OS, at risk individuals of lapatinib plus capecitabine induced nausea and vomiting should be closely monitored to minimise its potential occurrence - potentially via the addition of antiemetic prophylaxis.

The strengths of this study are the large sample size and the quality of data collected within the EMILIA trial. Further, the landmark analysis at 42 days provides the clinician with useful information early in treatment, thus potentially facilitating timely management to optimize patient outcomes. The results have also been confirmed in a sensitivity time-dependent Cox proportional hazards analysis. A potential study limitation is that clinical trials do not include all patients who may be treated in routine clinical care (i.e. strict inclusion criteria), and the EMILIA trial may not represent the most contemporary line of lapatinib plus capecitabine use in real-world practice. Thus, future studies will have a role in investigating the identified associations within a real-world cohort of ABC patients treated with lapatinib plus capecitabine. It is of interest to quantify the impact of the clinical trial setting as compared to routine clinical practice on the identified associations. Further, future studies should investigate the associations between lapatinib or capecitabine induced AE and outcomes when used in different settings, for example, early rash developed secondary to lapatinib in the adjuvant/neoadjuvant setting is approximately 50% which is significantly higher than herein 10, 15.

While lapatinib plus capecitabine-induced rash and hand foot syndrome was significantly associated with improved OS, and nausea and vomiting with worse OS; the associations of these AE with PFS were not statistically significant. The reason for this discrepancy is unclear, however PFS is an imperfect surrogate marker, which may be affected by variability in timing of assessments, investigators and measurement biases 23, 24. Further, assessed association with OS may be confounded by drug cessation, crossover or subsequent therapies 23, 24. An appreciation of these points indicates that the identified associations may not be isolated to lapatinib plus capecitabine. Rather they may represent individuals with greater sensitivity to anti-cancer medicines - which compounds to significant OS differences (appreciating that there were consistent trends, but not statistically significant associations, identified with PFS for lapatinib plus capecitabine induced-rash, hand-foot syndrome and nausea and vomiting).

In conclusion, ABC patients who experienced rash and hand-foot syndrome in the first 42 days of lapatinib plus capecitabine therapy had improved OS within EMILIA. This finding suggests that early rash or hand-foot syndrome could function as surrogate markers of therapeutic efficacy, and thus dose-escalation to grade 1 or 2 rash or hand-foot syndrome may translate into improved clinical outcomes in ABC patients treated with lapatinib plus capecitabine. Conversely, ABC patients who experienced nausea and vomiting within the first 42 days of lapatinib plus capecitabine therapy had worse OS, indicating that close monitoring of individuals at a high risk of lapatinib plus capecitabine induced nausea and vomiting is advisable.

## Figures and Tables

**Figure 1:**
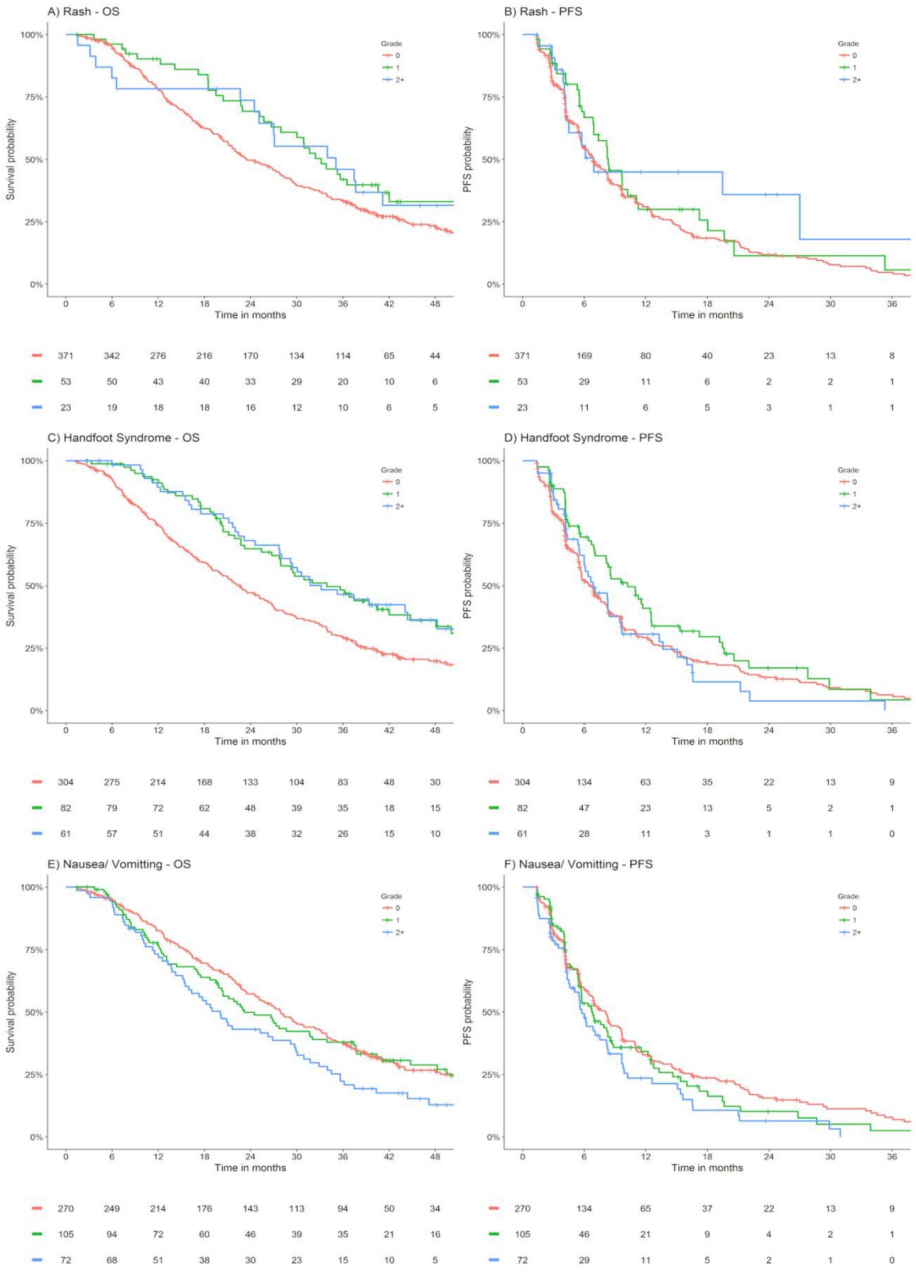
OS and PFS stratified by maximum grade of rash, hand foot syndrome, nausea/ vomiting within the first 42 days of lapatinib plus capecitabine treated ABC patients from EMILIA. Abbreviations: OS - overall survival, PFS - progression free survival.

**Table 1:** Summary of participant characteristics in the EMLIA analysis dataset

Summary of patient characteristics	Total Number 488
**The actual treatment given**	488 (100%)
**Age (years)**	53 (46 - 61)
**Race**	
White	367 (75%)
Asian	85 (17%)
Black or African American	21 (4%)
American Indian or Alaska Native	7 (1%)
Native Hawaiian or Other Pacific Islander	3 (1%)
Other	5 (1%)
**Race**	
Non-White	121 (25%)
White	367 (75%)
**Race**	
Non-Asian	403 (83%)
Asian	85 (17%)
**Weight (Kg)**	
Median (IQR)	68 (59 - 79)
Missing	9 (2%)
**ECOG Performance status**	
0	308 (63%)
1	172 (35%)
Missing	8 (2%)
**Visceral disease site at baseline**	328 (67%)
**Months from initial diagnosis**	
Median (IQR)	40 (18 - 77)
Missing	2 (0%)
**Progesterone Receptor Status**	
Positive	169 (35%)
Negative	304 (62%)
Missing	15 (3%)
**Estrogen Receptor Status**	
Positive	244 (50%)
Negative	237 (49%)
Missing	7 (1%)
**HER2 Status Fluorescence In-Situ Hybridisation**	
Positive	438 (90%)
Negative	6 (1%)
Missing	44 (9%)
**HER2 Status Immunohistochemistry**	
0+	2 (0%)
1+	2 (0%)
2+	54 (11%)
3+	429 (88%)
Missing	1 (0%)
**Any prior trastuzumab all settings**	488 (100%)
**Any prior anthracycline all settings**	297 (61%)
**Any prior taxane all settings**	486 (100%)

Data are median (IQR) or number of patients (%). Abbreviations: ECOG - Eastern Cooperative Oncology Group, IQR - Interquartile Range, HER2 - Human Epidermal Growth Factor Receptor 2, Kg - Kilogram

**Table 2:** Summary of maximum grade of AE within the first 42 days of lapatinib plus capecitabine, and within the entire follow up period

	First 42 days	Entire follow up
Rash*		
Grade 0	402 (82%)	342 (70%)
Grade 1	58 (12%)	83 (17%)
Grade 2+	28 (6%)	63 (13%)
**Hand Foot Syndrome**		
Grade 0	336 (69%)	197 (40%)
Grade 1	89 (18%)	66 (14%)
Grade 2+	63 (13%)	225 (46%)
**Nausea and Vomiting**		
Grade 0	290 (59%)	218 (45%)
Grade 1	114 (23%)	148 (30%)
Grade 2+	84 (17%)	122 (25%)
**Diarrhoea**		
Grade 0	167 (34%)	99 (20%)
Grade 1	148 (30%)	146 (30%)
Grade 2+	173 (35%)	243 (50%)
**Decreased Appetite**		
Grade 0	404 (83%)	371 (76%)
Grade 1	59 (12%)	78 (16%)
Grade 2+	25 (5%)	39 (8%)
**Gastrointestinal Inflammation#**		
Grade 0	372 (76%)	336 (69%)
Grade 1	76 (16%)	89 (18%)
Grade 2+	40 (8%)	63 (13%)
**Fatigue and Asthenia**		
Grade 0	359 (74%)	269 (55%)
Grade 1	81 (17%)	112 (23%)
Grade 2+	48 (10%)	107 (22%)

Data are number of patients (%). * - combined variable of preferred terms rash, exfoliative rash, rash pruritic, rash macular, rash erythematous, rash pustular, rash maculo-papular, rash papular, rash vesicular, and rash generalized. # - combined variable of preferred terms mucosal inflammation and stomatitis

**Table 3:** Summary of association between maximum grade of AE, OS and PFS within the first 42 days of lapatinib plus capecitabine therapy

		OS		PFS	
Variable	N	HR [95% CI]	*p*	HR [95% CI]	*p*
**Rash**			0.046		0.197
Grade 0	371	1.00		1.00	
Grade 1	53	0.67 [0.46-0.98]		0.82 [0.58-1.16]	
Grade 2+	23	0.71 [0.42-1.19]		0.68 [0.40-1.17]	
**Hand Foot Syndrome**			<0.001		0.106
Grade 0	304	1.00		1.00	
Grade 1	82	0.58 [0.43-0.80]		0.75 [0.56-1.00]	
Grade 2+	61	0.61 [0.43-0.86]		1.04 [0.75-1.44]	
**Nausea and Vomiting**			0.027		0.084
Grade 0	270	1.00		1.00	
Grade 1	105	1.08 [0.82-1.42]		1.11 [0.85-1.45]	
Grade 2+	72	1.52 [1.13-2.03]		1.41 [1.05-1.90]	
Diarrhoea			0.142		0.539
Grade 0	153	1.00		1.00	
Grade 1	138	0.80 [0.61-1.05]		0.87 [0.67-1.14]	
Grade 2+	156	0.79 [0.60-1.02]		0.89 [0.69-1.15]	
**Decreased Appetite**			0.603		0.787
Grade 0	370	1.00		1.00	
Grade 1	57	0.98 [0.70-1.38]		1.04 [0.74-1.45]	
Grade 2+	20	0.75 [0.42-1.34]		1.22 [0.70-2.13]	
**Gastrointestinal Inflammation**			0.169		0.17
Grade 0	340	1.00		1.00	
Grade 1	71	0.74 [0.53-1.02]		0.77 [0.56-1.06]	
Grade 2+	36	0.94 [0.63-1.41]		0.80 [0.53-1.20]	
**Fatigue and Asthenia**			0.105		0.54
Grade 0	330	1.00		1.00	
Grade 1	75	0.73 [0.53-1.00]		0.92 [0.68-1.23]	
Grade 2+	42	1.08 [0.75-1.56]		0.83 [0.57-1.20]	

Abbreviations: CI - confidence interval, HR - hazard ratio, OS - overall survival, PFS - progression free survival, N - Number

**Table 4:** Summary of the association between grade of rash, hand-foot syndrome and nausea plus vomiting with OS in time-dependent Cox proportional hazard regression analysis

	Univariable analysis			Adjusted analysis	
Variable	HR [95% CI]	p		HR [95% CI]	p
**Rash**		<0.001			<0.001
Grade 0	1.00			1.00	
Grade 1	0.51 [0.37-0.71]			0.52 [0.37-0.72]	
Grade 2+	0.65 [0.46-0.92]			0.62 [0.43-0.89]	
**Hand Foot Syndrome**		<0.001			<0.001
Grade 0	1.00			1.00	
Grade 1	0.63 [0.45-0.88]			0.57 [0.40-0.80]	
Grade 2+	0.56 [0.45-0.71]			0.53 [0.42-0.69]	
**Nausea and Vomiting**		0.044			0.134
Grade 0	1.00			1.00	
Grade 1	1.00 [0.77-1.29]			0.95 [0.73-1.24]	
Grade 2+	1.37 [1.05-1.78]			1.26 [0.96-1.66]	

## Abbreviations

AE: adverse events
PFS: progression-free survival
OS: overall survival
HER2: human epidermal growth factor receptor 2
ABC: advanced breast cancer
HR: Hazard Ratio
CI: Confidence Interval
EGFR: epidermal growth factor receptor
mRECIST: modified Response Evaluation Criteria in Solid Tumours version
CTCAE: Common Terminology Criteria for Adverse Events
